# Current impacts of elevated CO_2_ on crop nutritional quality: a review using wheat as a case study

**DOI:** 10.1007/s44154-025-00217-w

**Published:** 2025-05-08

**Authors:** Jiata Ugwah Ekele, Richard Webster, Fatima Perez de Heredia, Katie E. Lane, Abdulmannan Fadel, Rachael C. Symonds

**Affiliations:** 1 School of Biological and Environmental Sciences, Liverpool John Moores University, Byrom Street, Liverpool, L3 3AF UK; 2 Institute of Health Research, Liverpool John Moores University, Liverpool, UK; 3 Liverpool Centre for Cardiovascular Science, Liverpool John Moores University, Liverpool, UK; 4 Department of Sports and Exercise Sciences, Liverpool John Moores University, Byrom Street, Liverpool, L3 3AF UK; 5 Department of Nutrition and Health, College of Medicine and Health Sciences, United Arab Emirates University, P.O. Box 1555, Al Ain, United Arab Emirates

**Keywords:** Elevated carbon dioxide, eCO_2_, Crop nutrients, Photosynthesis, Nutrition, Climate change, Food security, Wheat

## Abstract

This review synthesises current research findings and modelling approaches to explore the impact of elevated atmospheric carbon dioxide (eCO_2_) concentrations on crop productivity, water and nutrient use efficiency, plant nutritional quality, and the implications for global food security. Over recent decades, rising atmospheric CO_2_ levels have sparked significant concern due to their role in driving climate change. While some studies highlight the potential benefits of eCO_2_, such as increased crop yields and improved water-use efficiency, many recent investigations reveal a concerning decline in crop nutritional quality. eCO_2_ has been shown to reduce concentrations of key nutrients, including nitrogen, minerals, vitamins, polyphenols, and other non-nutrient compounds, as well as alter gene expression. These changes are further complicated by interactions with heat stress and drought, presenting significant challenges in predicting sustainable future crop productivity. These nutritional declines exacerbate the global crisis of malnutrition and hidden hunger, threatening the achievement of Sustainable Development Goal 2 (SDG2), which aims to end hunger and ensure food security. Addressing these challenges requires further research, interdisciplinary collaboration, and innovative approaches to mitigate the adverse effects of eCO_2_ on crop physiology and nutritional content while maximising agricultural sustainability. This review aims to provide insights into the complex mechanisms governing crop responses to eCO_2_ using wheat as a model and proposes pathways for future research and agricultural practices. These strategies are critical for tackling the intricate dynamics of climate variability, ensuring nutrient-rich food production, and securing food security in the face of a rapidly changing climate.

**Supplementary Information:**

The online version contains supplementary material available at 10.1007/s44154-025-00217-w.

## Introduction

Since the industrial revolution, atmospheric CO_2_ concentrations have increased from approximately 200 ppm to over 400 ppm due to anthropogenic activities such as fossil fuel combustion and deforestation (Shah et al. 2024). Current projections suggest that, if emissions continue unabated, atmospheric CO_2_ levels could reach 550 ppm by 2050, potentially doubling pre-industrial concentrations (IPCC 2021; GISTEMP 2019). This anticipated rise in CO_2_ has far-reaching implications for global ecosystems, climate, and agriculture as it influences not only the photosynthetic processes that fuel plant growth but also interacts with environmental stressors like drought and heat in ambiguous ways. Research has shown that elevated CO_2_ (eCO_2_) can enhance crop photosynthesis and water-use efficiency, leading to potential increases in yields under favourable conditions (Jin et al. 2019). However, several studies indicate that these yield improvements are not without underlying plant nutrient trade-offs, as the broader impacts of eCO_2_ on crop nutritional quality and long-term adaptation have raised concerns about food security and public health (Chang et al. 2023).

**Fig. 1:**
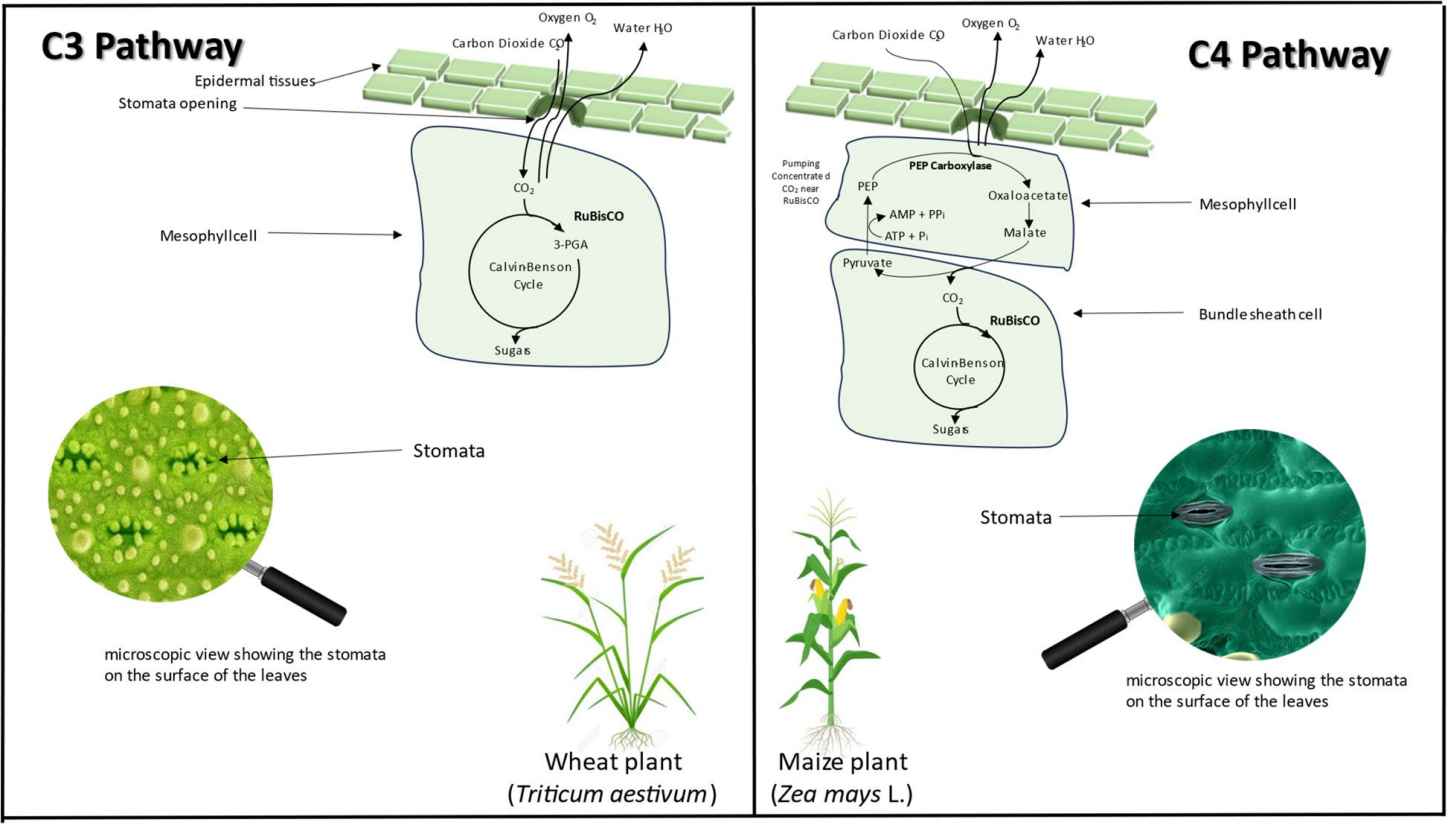
Comparison of C3 and C4 photosynthetic pathway—in C3 plants, CO_2_ fixation occurs directly via RuBisCO, forming a three-carbon compound in the Calvin cycle while C4 plants employ an initial CO_2_ fixation step in mesophyll cells, producing a four-carbon compound that is transported to bundle sheath cells, and CO_2_ is concentrated around RuBisCO to enhance photosynthetic efficiency and reduce photorespiration

Photosynthesis, the process by which plants transform light energy into chemical energy, necessitates the presence of CO_2_ (Siddiqui et al. 2018). The enzyme ribulose-1,5-bisphosphate carboxylase/oxygenase (RuBisCO) fixes CO_2_ in the Calvin–Benson–Bassham (CBB) cycle, in the stroma of chloroplasts, to generate organic molecules, primarily sugars and also a range of other carbohydrates, which plants utilise as structural elements and sources of energy (Siddiqui et al. 2018). The precise reaction in which CO_2_ is fixed into an organic molecule is used to classify plants into C3/C4 groups. In C3 plants, such as wheat and rice, CO_2_ is directly fixed into a three-carbon compound called 3-phosphoglycerate during photosynthesis, while C4 plants like maize and sugarcane employ an additional biochemical pathway to concentrate CO_2_ in specialised bundle sheath cells, enhancing carbon assimilation and water use efficiency under low CO_2_ or high-temperature conditions (Fig. 1) (Semba et al. 2022). Thus, the increase in atmospheric CO_2_ levels has been associated with higher photosynthetic rates, particularly in C3 plants, contributing to enhanced biomass production and sugar accumulation in some food crops under optimal free-air CO_2_ enrichment (FACE) and closed environment conditions (Cai et al. 2016).

Nonetheless, the relationship between eCO_2_ and crop performance is more intricate, especially when considering the interaction between eCO_2_ and other environmental stressors, such as heat and drought. While eCO_2_ can initially stimulate photosynthesis, its benefits may be mitigated or negated by these concurrent stress factors (Taub and Wang 2008). For instance, heat stress reduces RuBisCO efficiency in C3 plants, limiting the potential yield gains from eCO_2_ (Furbank 2016). Likewise, drought-induced reductions in stomatal conductance restrict CO_2_ uptake, further curbing photosynthetic efficiency and growth (Kumar and Verma 2018). Although C4 plants are generally more resilient to heat and drought due to their carbon-concentrating mechanisms, extreme environmental conditions can still impair their productivity by disrupting the Calvin cycle and reducing electron transport efficiency (Jin et al. 2019). These complexities make it challenging to predict crop responses to eCO_2_ in real-world scenarios, where multiple stressors are often present.

More critically, the rising concern is the negative impact of eCO_2_ on crop nutritional quality. Recent studies have shown that eCO_2_ reduces the concentration of essential micronutrients such as zinc, iron, and protein in many staple crops, exacerbating the global issue of hidden hunger—the insufficient intake of essential nutrients despite adequate caloric consumption (Myers et al. 2014; Loladze 2014). For instance, wheat grown under eCO_2_ conditions has shown protein reductions of up to 65% (Myers et al. 2014), while declines of over 50% in zinc and iron concentrations have been observed in rice and other staple crops (Loladze 2014). Also, studies by Taub and Wang (2008) highlighted changes in the expression of genes and metabolic pathways in response to elevated CO_2_ levels, further elucidating the molecular mechanisms underlying these changes. These findings underscore the exacerbation of the global crisis of malnutrition due to diminished crop nutritional quality under eCO_2_ conditions, posing severe challenges for food security and public health. The reduction in micronutrient content poses severe risks to global food security, particularly in developing regions that rely heavily on these crops as primary dietary sources of nutrition. The long-term implications of such declines could exacerbate malnutrition and weaken immune defences, especially in vulnerable populations.

Moreover, the increase in carbohydrate production under eCO_2_, while potentially beneficial for yield, has been linked to negative health outcomes. Elevated levels of sugars in staple crops may contribute to the rising incidence of diet-related diseases such as diabetes and obesity, particularly in developed countries where caloric intake is already high (Sparling et al. 2024). In regions such as the United States, where food consumption patterns are consistent, the nutrient-diluting effect of eCO_2_ could result in individuals consuming the same amount of food but with a greater proportion of low-nutrient carbohydrates, which may contribute to the prevalence of metabolic disorders and exacerbate existing public health challenges (Raiten and Bremer 2023). This shift not only highlights the agricultural risks posed by eCO_2_ but also underscores the broader health implications of a changing global food system.

As such, the intersection of eCO_2_ with environmental stressors and its cascading effects on both agricultural productivity and nutritional quality calls for a comprehensive re-evaluation of how future food systems will be managed. This review synthesises current knowledge on the physiological and molecular mechanisms underlying plant responses to eCO_2_, with a particular focus on how these changes influence crop yields and nutrient content in the context of heat and drought stress. It further explores the broader implications for global food security and public health, identifying strategies to mitigate the risks associated with nutrient dilution and offering directions for future research to enhance resilience in agricultural systems.

## The trends of increasing atmospheric CO_2_ concentrations (eCO_2_)

**Fig. 2:**
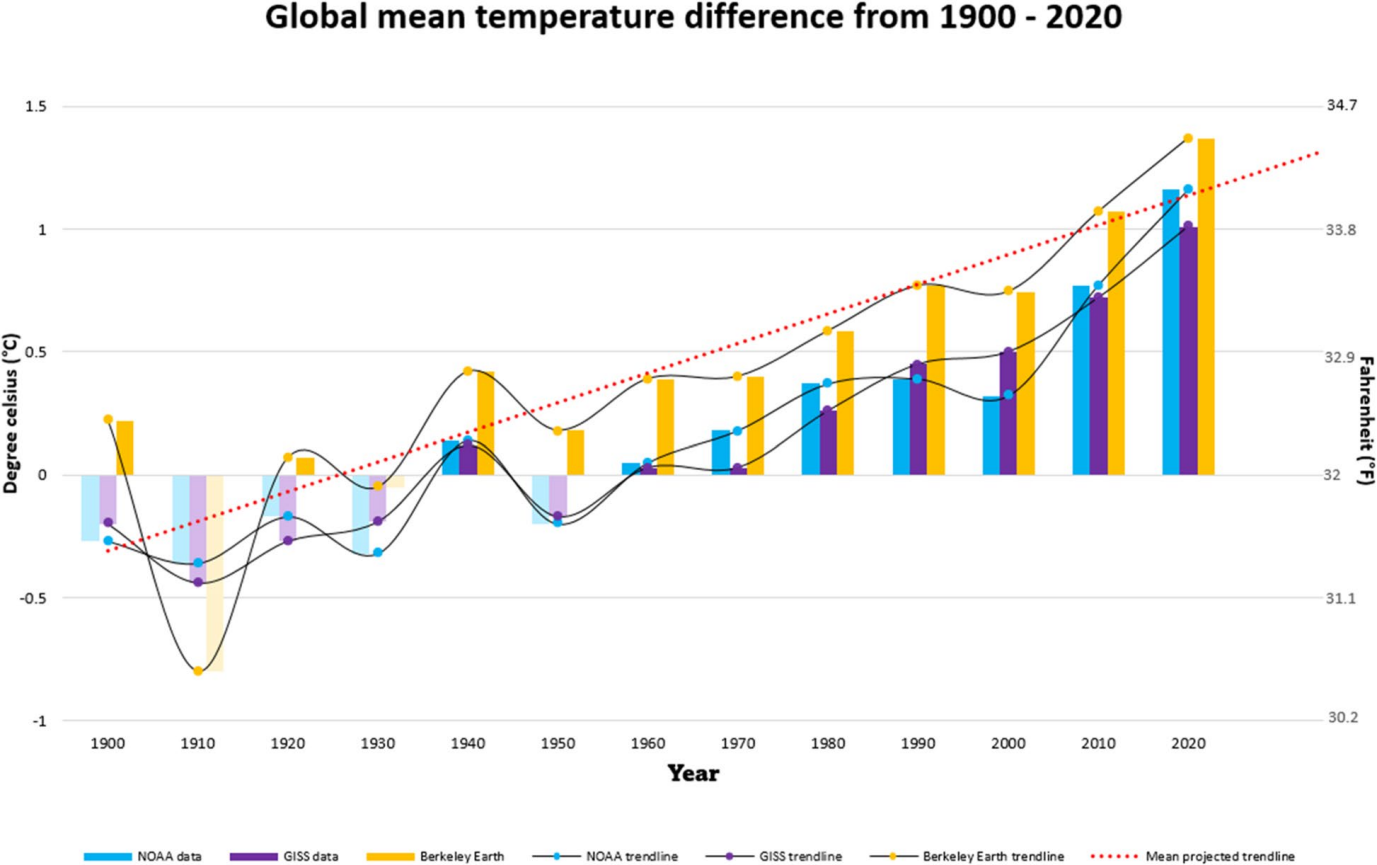
Bar plot showing global mean temperature difference from 1900 to 2020. Data culled from the National Oceanic and Atmospheric Administration (NOAA), NASA’s Goddard Institute for Space Studies (GISS) and the Berkeley Earth observation dataset (2023) (GISTEMP 2019; Lenssen et al. 2019; Rohde et al. 2013; Zhang et al. 2019)

In concordance with atmospheric CO_2_ concentrations, mean decadal surface temperatures have increased globally from 1900 to 2020 (Fig. 2). Direct measurements of CO_2_ obtained from monitoring stations worldwide provide compelling evidence of this increase, primarily attributed to human activities. Ice core records and monitoring stations such as Hawaii’s Mauna Loa Observatory and the Keeling Curve of Scripps Institution of Oceanography have monitored CO_2_ levels since the late 1950s, revealing a continuous upward trend with annual average rates exceeding two parts per million (ppm) (Janssens-Maenhout et al. 2020). More recent ice core records spanning the past few centuries corroborate the unprecedented rise in CO_2_ concentrations since the onset of the industrial revolution, with CO_2_ concentrations in the atmosphere surpassing 400 ppm in recent decades (Eldering et al. 2017). Furthermore, Satellite observations, particularly from missions like NASA’s Orbiting Carbon Observatory-2, complement ground-based measurements, offering valuable insights into spatial and temporal CO_2_ variability (Crisp et al. 2017).

The adverse impact of climate change on various components of the ecosystem is well documented. The rise in the Earth’s surface temperature has led to shifts in precipitation patterns, ecosystem alterations, and habitat loss for numerous species (IPCC 2021). Rising sea levels, mainly due to the thermal expansion of saltwater and melting ice caps, pose significant threats to infrastructure and coastal populations, especially in low-lying areas (IPCC 2021). Flooding, erosion, and saltwater intrusion are causing displacement of populations, loss of agricultural land, and degradation of freshwater resources, particularly affecting small island nations and densely populated coastal cities (Campbell-Lendrum et al. 2023). Climate change also intensifies extreme weather events such as heatwaves, floods, droughts, and tropical cyclones, causing immediate damage to infrastructure and livelihoods. Vulnerable populations, including the poor, elderly, and marginalised communities, suffer disproportionately from these events, exacerbating social inequalities (IPBES 2019). Moreover, IPCC (2021) reports that climate change disrupts ecological systems worldwide, altering the distribution/abundance of species and impacting ecosystem composition and function with the biodiversity of coral reefs, mangrove forests, and polar ecosystems most particularly vulnerable.

## eCO_2_: how does it affect plants

### Photosynthesis

Research spanning several decades has elucidated the physiological mechanisms underlying the response of crops to elevated CO_2_, particularly photosynthesis (Semba et al. 2022). CO_2_ is a fundamental substrate in photosynthesis, the essential process by which plants harness sunlight to convert CO_2_ and water into glucose and oxygen, utilising ATP (adenosine triphosphate) and NADPH (nicotinamide adenine dinucleotide phosphate) as energy and reducing power sources, respectively (Lenka et al. 2021). This occurs in the CBB cycle, which begins with the carboxylation of ribulose-1,5-bisphosphate (RuBP) by RuBisCO enzyme (Siddiqui et al. 2018). The carboxylation process produces 3-phosphoglycerate, which is used in two reactions to generate triose phosphates, glyceraldehyde phosphate and dihydroxyacetone phosphate (Raines 2003). Most of these triose phosphates are utilised to regenerate RuBP, while some are converted into sucrose and starch, crucial carbon compounds for plant growth and development (Raines 2003). The Calvin cycle also provides intermediates for various metabolic pathways in the chloroplast, including isoprenoid biosynthesis, the shikimate pathway, and precursors for cell wall formation and nucleotide metabolism (Kumar and Verma 2018).

C4 plants differ from C3 plants in that they possess a biochemical mechanism that concentrates CO_2_ in bundle sheath cells, enhancing photosynthetic efficiency and minimising photorespiration (Cai et al. 2016). In C4 plants, atmospheric CO_2_ is first fixed in mesophyll cells by phosphoenolpyruvate carboxylase (PEPC) to form a four-carbon compound, typically oxaloacetate, which is then converted to malate and transported to bundle sheath cells. Within these cells, malate is decarboxylated to release CO_2_, thus increasing CO_2_ concentration around RuBisCO, optimizing photosynthesis in the Calvin cycle (Zhao et al. 2017). C3 plants are generally more responsive to atmospheric CO_2_ concentration changes than C4 plants due to the absence of a carbon-concentrating mechanism (Fig. 1).

Numerous studies have demonstrated that eCO_2_ typically stimulates photosynthesis in C3 plants, primarily through increased carboxylation rates and reduced photorespiration. As CO_2_ concentration rises, the partial pressure of CO_2_ at the site of RuBisCO activity increases, enhancing the carboxylation efficiency of the enzyme. This phenomenon, known as CO_2_ fertilisation, leads to higher carbon fixation rates and increased biomass production in C3 plants under eCO_2_ conditions (Jin et al. 2019). However, the magnitude of this enhancement varies based on several factors, including species-specific responses, nutrient availability, and environmental conditions. Research suggests that the stimulatory effect of eCO_2_ may diminish over time as plants acclimate to prolonged exposure (Lenka et al. 2021). Additionally, the availability of essential resources, such as nitrogen and water, can modulate the response of C3 plants to eCO_2_, potentially offsetting its positive effects on photosynthesis (Abdelhakim et al. 2022).

While C4 plants generally exhibit higher photosynthetic efficiency than C3 plants under current atmospheric CO_2_ levels, their response to eCO_2_ remains a contentious topic of research. Unlike C3 plants, which typically show a stimulatory response to increased CO_2_, the impact of eCO_2_ on C4 plants is less straightforward and can vary depending on the species and surrounding ecological conditions (Panozzo et al. 2019). Some studies have reported negligible or even negative impacts of eCO_2_ on photosynthetic activities in C4 plants, attributing this to their already efficient CO_2_-concentrating mechanisms (Panozzo et al. 2019). Moreover, the potential downregulation of PEPC activity in response to eCO_2_ may limit further enhancement of photosynthesis in C4 species (Siddiqui et al. 2018). However, other research indicates that certain C4 species may still benefit from eCO_2_, exhibiting enhanced biomass production and water use efficiency (WUE) under favourable conditions (Shah et al. 2024).

Several factors contribute to the variability in the observed responses of C4 plants to eCO_2_. Environmental conditions such as temperature, water availability, and nutrient levels play a significant role in mediating plant responses to eCO_2_. In many experimental studies, some optimal growing conditions are standardised, which do not reflect the variability and extremity of real-world environments (Chavan et al. 2019). For example, maize (*Zea mays*), a major C4 crop, has shown minimal photosynthetic enhancement under eCO_2_ due to its PEPC mechanism (Ziska 2022). Conversely, certain studies indicate that maize may benefit from eCO_2_ under water-limited conditions, where improved WUE leads to higher biomass accumulation (Shah et al. 2024). Species like *Sorghum bicolor* and certain grasses in arid or semi-arid ecosystems may experience increased biomass and WUE under eCO_2_, especially when other resources such as water and nutrients are not limiting (Yilmaz et al. 2017). Some studies report modest increases in growth and WUE in sugarcane under specific environmental conditions (Fitzgerald et al. 2016), particularly in water-limited scenarios where stomatal closure reduces water loss. These responses highlight the influence of ecological conditions, such as water availability and nutrient status, in modulating the impact of eCO_2_ on C4 plants.

Differences in experimental designs, such as growth chambers versus open-field trials, duration of exposure to elevated CO_2_, and the inclusion of additional stress factors, can lead to divergent results across studies. Laboratory settings often isolate eCO_2_ as a variable, while field studies incorporate the complex interactions of eCO_2_ with temperature, water, and soil factors, leading to more nuanced and often less favourable outcomes.Furthermore, genetic and physiological diversity within and across species leads to differences in how individual cultivars or varieties respond to eCO_2_. For example, different maize genotypes exhibit varying degrees of responsiveness to eCO_2_, with some showing no significant growth improvements (Zhao et al. 2017).

Legumes are uniquely positioned to optimise the benefits of eCO_2_ by coordinating enhanced nitrogen fixation with increased photosynthesis, unlike many non-leguminous C3 plants (Rogers et al. 2009). For instance, FACE studies on soybean and clover have shown that legumes can enhance nitrogen fixation under eCO_2_ conditions, alongside increases in photosynthetic carbon absorption and sugar levels in leaves (Ziska 2022). Notably, the results of the potential benefits of eCO_2_ from these FACE experiments do not account for other climate change interactions (such as drought and heat) that exist in the real world. Furthermore, eCO_2_ may protect nitrogen fixation from moderate drought by reducing stomatal conductance, thereby lowering canopy transpiration and facilitating moisture translocation from the soil to plants, including soybeans (Abdelhakim et al. 2022). This mechanism can help prevent or delay reductions in nitrogen fixation during periods of moderate drought stress (Shah et al. 2024). Consequently, as atmospheric CO_2_ levels rise alongside changes in rainfall patterns, eCO_2_ may provide some protection against drought-induced declines in nitrogen fixation (Furbank 2016).

Elevated CO_2_ have been reported to upset the balance between carbon and nitrogen in crops which is fundamental to their growth and nutrient quality as these elements are essential for synthesisng structural, metabolic and storage compound (Jauregui et al. 2015). As eCO_2_ increases carbon fixation and RuBisCO activity, there happens to be greater accumulation of carbohydrates in plant tissues, and this carbon-rich environment disrupts the carbon/nitrogen (C/N) balance by diluting nitrogen-based compounds, especially when nitrogen availability is limited in the soil (Jin et al. 2019). Consequently, a lower nitrogen concentration can reduce the nutritional quality of crops, impacting protein content and mineral levels in edible tissues because nitrogen is a key component of amino acids, nucleotides, and chlorophyll (Lenka et al. 2021).

Finally, alterations in gene expression related to nitrogen and carbon metabolism, photosynthesis, and stress responses have been documented in crops exposed to eCO_2_. For example, Ainsworth and Long (2005) found that elevated CO_2_ can upregulate genes involved in carbon fixation while downregulating those associated with nitrogen assimilation in soybeans. These gene expression changes can significantly impact crop growth, yield, and nutritional quality, with observed responses across various staple crops, including maize, rice, and wheat (Jin et al. 2019).

### Crop Water Use Efficiency (WUE) and yields

Elevated CO_2_ has been associated with improvements in both crop WUE and yields, though the effects are complex and context-dependent. Several mechanisms have been described through which eCO_2_ levels can improve WUE and, consequently, crop yield. Elevated CO_2_ levels have been shown to induce stomatal closure in many plant species, reducing transpiration rates and enhancing WUE. For instance, FACE experiments with wheat (*Triticum aestivum*) and soybean (*Glycine max*) demonstrated increased WUE under eCO_2_ conditions, as stomatal closure reduced water loss while photosynthesis was maintained (Lenka et al. 2021). In addition, controlled-environment studies on C3 plants such as rice (*Oryza sativa*) and barley (*Hordeum vulgare*) have shown similar improvements in WUE (Ainsworth and Long 2005). These findings suggest that eCO_2_ could enhance crop resilience, particularly under water-limited conditions in drought-prone areas, by allowing plants to conserve water while maintaining high photosynthetic rates (Siddiqui et al. 2018).

Increased CO_2_ concentrations can also stimulate root growth and development, leading to enhanced water uptake. Studies indicate that eCO_2_ promotes the proliferation of fine roots, increasing the surface area available for water absorption. For example, FACE experiments on cotton (*Gossypium hirsutum*) showed a significant increase in fine root length density, which improved water acquisition from deeper soil layers under eCO_2_ (Kubisch et al. 2016). Additionally, eCO_2_ can enhance the effectiveness of water use within plant tissues by promoting the synthesis of osmoprotectants like proline and antioxidants such as superoxide dismutase, which help plants tolerate water stress and maintain cellular function (Shah et al. 2024). These compounds play a critical role in mitigating oxidative damage and ensuring metabolic stability during drought conditions, further contributing to WUE in crops grown under eCO_2_ conditions.

Moreover, the impact of eCO_2_ extends to the interrelationships between soil microbes and crops, which play a critical role in nutrient cycling and water availability. Research has shown that eCO_2_ can modify the structure and reactivity of soil microbial populations, enhancing nutrient mineralisation and organic matter decomposition (Zhao et al. 2017). These microbial-soil interactions can indirectly improve crop WUE by facilitating nutrient uptake and enhancing soil water retention, thereby supporting plant growth and productivity. However, the long-term implications of such microbial activity are complex. While short-term benefits include increased nutrient cycling, prolonged microbial activity might deplete essential nutrients in already nutrient-limited soils, diminishing long-term soil fertility and potentially increasing greenhouse gas emissions through enhanced microbial respiration (Perkowski et al. 2024). Gradual increases in atmospheric CO_2_ may allow microbial communities to adapt over time, potentially muting some of the rapid changes observed in experimental setups (Classen et al. 2015).

Despite these potential benefits to WUE, real-world conditions often limit the advantages observed in controlled environments. In reality, drought conditions often coincide with heat stress, which can exacerbate the negative impacts on crop physiology and negate the advantages of stomatal closure. For example, even with reduced water loss due to stomatal closure under high temperatures, plants may still experience heat-induced damage to photosynthetic machinery, leading to decreased productivity (Okolie and Ogundeji 2022). Furthermore, the reduction in transpiration also limits evaporative cooling, which can cause canopy temperatures to rise, intensifying heat stress and limiting the overall benefit of improved water uptake, further reducing crop yields (Taub and Wang 2008; Prakash et al. 2017). Additionally, the extent of root proliferation under eCO_2_ varies between species; while some species, such as cotton and wheat, exhibit enhanced root growth, others may show limited or inconsistent responses depending on their genetic makeup and environmental conditions (Sayed et al. 2022). In addition, the co-occurrence of eCO_2_ with heat stress can impair root function; high temperatures can damage root structures and inhibit the plant’s ability to access deeper soil moisture reserves, reducing the capacity for water uptake despite increased root proliferation (Semba et al. 2022).

In parallel, eCO_2_ influences crop yield responses across different species and conditions. Evidence from experimental studies, particularly FACE experiments and controlled environments, indicate yield increase for crops grown under eCO_2_ (Jin et al. 2019). A comprehensive meta-analysis of FACE experiments on rice, soybeans, and wheat consistently revealed yield increases under eCO_2_ conditions across diverse geographic regions (Ziska 2022). Specifically, research focusing on C3 crops, particularly wheat and soybeans, has shown significant enhancements in biomass yield and grain output in response to eCO_2_ levels (Semba et al. 2022). However, in the case of C4 crops, such as sorghum and maize, studies suggest that while yield increases may be smaller compared to C3 crops, these species still benefit from improved water and nitrogen use efficiency under eCO_2_ conditions (Cai et al. 2016). This nuanced response indicates that even within the C4 group, the benefits of elevated CO_2_ are context-dependent.

Yet, the positive results observed in FACE experiments and controlled environment studiesdo not fully replicate the complexities of real-world agricultural conditions where multiple stressors such as heat, drought, pest pressures, and nutrient limitations co-occur. FACE experiments, while valuable for simulating future atmospheric CO_2_ levels, often lack the full complexity of natural environments which may undermine yield gains in field conditions. For instance, drought and heat stress, which frequently accompany elevated CO_2_ in many regions, have been reported to negate the yield benefits observed under experimental conditions (Jin et al. 2019). Besides, FACE experiments and such likes, do not account for soil degradation, nutrient depletion, or varying agricultural practices that affect crop productivity in natural farming systems. Hence, this disconnect raises concerns about the scalability and applicability of the observed benefits of eCO_2_ to global food production, where environmental interactions and resource constraints complicate crop responses.

Modelling approaches have also been utilised to assess the long-term impacts of eCO_2_ on crop productivity (Chavan et al. 2019). These models integrate physiological processes, environmental variables, and agronomic practices to simulate crop growth and yield under varying CO_2_ scenarios. While there is variability among models regarding the magnitude of CO_2_-induced yield enhancements, most predict a potential benefit of eCO_2_ on crop yields, mainly when optimal management practices are employed. However, uncertainties remain about how additional factors—such as climate variability, nutrient availability, and pressures from pests or diseases—may influence crop responses to elevated CO_2_.

Although experimental evidence indicates that eCO_2_ can enhance crop yields under optimal growing conditions, the extent of this effect varies significantly across species and environmental contexts. Meta-analyses by Ainsworth and Rogers (2007) have reported that yield stimulations attributable to eCO_2_ ranged from 10 to 25% for C3 crops, with C4 crops demonstrating more modest responses. However, these estimates often fail to account for the complex interactions between eCO_2_ and other environmental stressors, such as heat and drought, which can significantly impact overall crop productivity. So, while the potential benefits of eCO_2_ on crop yields are supported by experimental and modelling studies, the interplay of multiple factors necessitates a cautious interpretation of these findings, particularly in real-world agricultural settings.

## eCO_2_: what does it mean to us?

### Counter interaction with heat stress and drought

**Fig. 3:**
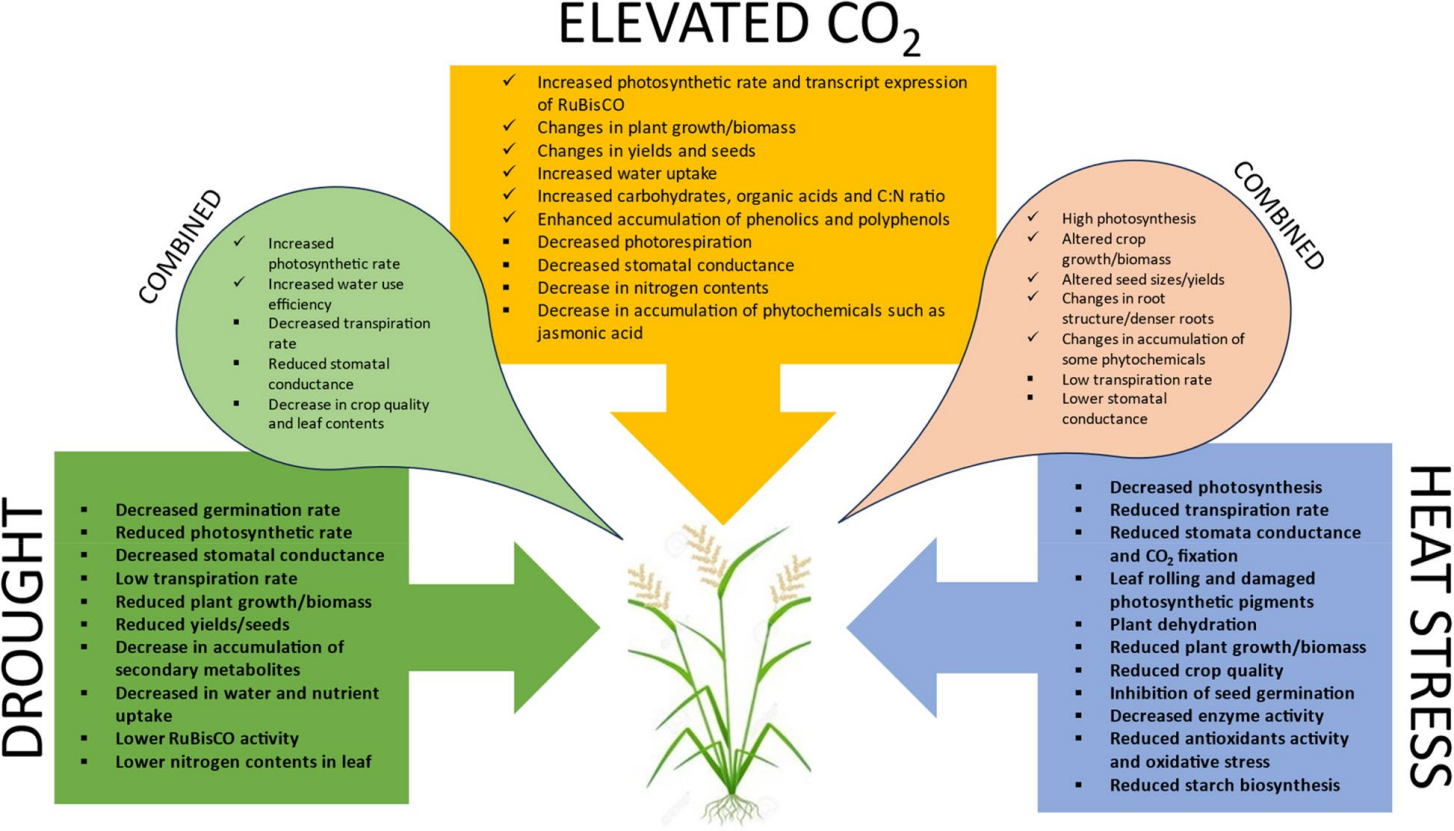
Summary of the effect of eCO2 on crops and its counter interactions with drought and heat stress

Heat stress presents a significant challenge to global crop productivity, severely disrupting physiological processes essential for growth and development (Fig. 3). Prolonged high temperatures can accelerate leaf senescence, reduce pollen viability, and impair photosynthetic efficiency, ultimately leading to considerable yield losses in many crops (Furbank 2016). Elevated temperatures increase the rate of evapotranspiration, thereby exacerbating water deficits and further reducing crop water status (Prakash et al. 2017). Moreover, the intensity and frequency of heat events are projected to increase under future climate scenarios, amplifying the detrimental effects of heat stress on crops and aggravating global food security concerns (Campbell-Lendrum et al. 2023).

The interaction between eCO_2_ and heat stress is complex and context-dependent, often challenging the simplified expectation that CO_2_-induced increases in crop yields can offset the negative effects of rising temperatures. While eCO_2_ has been shown to enhance WUE and mitigate oxidative stress by increasing antioxidant activity and reducing reactive oxygen species (ROS) damage (Zhao et al. 2017), these benefits may be limited under extreme heat conditions. The efficacy of eCO_2_ in counteracting heat stress depends on the severity and duration of heat events, as well as crop-specific physiological responses. Field studies on crops such as soybean and rice have demonstrated that although eCO_2_ can initially buffer against heat-induced yield reductions, this effect is often diminished or negated under prolonged and extreme heat, underscoring the need for more nuanced assessments of climate-crop interactions (Alsherif and AbdElgawad 2023).

Drought is another major threat to agriculture, with the frequency and severity of drought events expected to escalate in response to climate change (Chang et al. 2023). Water scarcity induces a series of physiological responses in plants, including stomatal closure, reduced cell expansion, decreased CO_2_ diffusion, and hormonal changes that activate drought-response pathways (Jin et al. 2019). These mechanisms, while essential for plant survival, often result in trade-offs that compromise yield under prolonged drought conditions. Stomatal closure, for example, limits CO_2_ uptake, reducing photosynthetic rates and, subsequently, crop biomass and yield (Shah et al. 2024). Meta-analyses by Zhao et al. (2017) have shown that even short periods of water deficit during the reproductive phase can lead to substantial yield losses, particularly in drought-sensitive crops such as maize and wheat. The compounding effects of heat stress often exacerbate the physiological stress caused by drought, amplifying the magnitude of yield reductions and further complicating adaptation strategies (Lenka et al. 2021).

Recent studies have increasingly highlighted that the response of crops to eCO_2_ is modulated by interactions with other environmental factors, including water availability, temperature, and nutrient status (Fitzgerald et al. 2016). As explained above, eCO_2_ can increase root elongation and enhance water absorption, potentially conferring partial resilience to drought stress (Campbell-Lendrum et al. 2023); however, when drought stress is coupled with heat stress, the synergistic effects can overwhelm these benefits, often resulting in significant net yield losses (Chavan et al. 2019). Rising temperatures further exacerbate the detrimental effects of water stress on photosynthesis by increasing photorespiration and reducing RuBisCO efficiency, especially in C3 crops such as wheat and rice (Furbank 2016). These physiological constraints are compounded by nutrient limitations—particularly nitrogen availability—that can restrict plants’ ability to fully capitalise on the stimulatory effects of eCO_2_ on photosynthesis (Fitzgerald et al. 2016).

Importantly, eCO_2_ does not uniformly benefit all crops or growing conditions. The co-occurrence of eCO_2_, heat, and drought stress triggers highly complex physiological responses in plants, often leading to non-additive and unpredictable effects on yields (Campbell-Lendrum et al. 2023). *Triticum aestivum*, as a case study, has shown increased biomass production and improved WUE under eCO_2_, but these gains are often diminished when combined with heat stress, which accelerates plant senescence and reduces grain filling (AbdElgawad et al. 2020). Similarly, *Zea mays* experiences only modest or negligible yield benefits under eCO_2_, and its sensitivity to drought and heat can drastically curtail any potential growth advantages. In *Oryza sativa*, eCO_2_ has been shown to enhance growth in controlled experiments, but real-world heat stress can lead to severe reductions in grain quality and nutritional value (Wang et al. 2022). Furthermore, modelling studies have shown that in certain scenarios, the combination of eCO_2_ and extreme heat can lead to net reductions in crop productivity due to impaired photosynthesis and reproductive failure (Chavan et al. 2019). These findings underscore the complexity of projecting future agricultural outcomes in a warming climate and highlight the need for integrated climate-crop models that account for multiple stressors.

Therefore, while eCO_2_ may offer some buffering capacity against heat and drought stress, these benefits are highly conditional and often overridden by the intensifying effects of global climate change. Understanding the intricate interplay between CO_2_ levels, temperature, water availability, and nutrient status is critical for developing adaptive agricultural strategies that can sustain productivity in the face of climate-induced challenges. Future research should focus on refining models of crop responses to multiple stressors and identifying crop varieties with enhanced resilience to the combined effects of heat, drought, and eCO_2_.

### Implications for future agricultural productivity

The interactive effects of eCO_2_, drought, and heat stress alone pose considerable challenges to global agricultural productivity. The combination of these stressors has been shown to lead to significant yield losses across a wide range of crops, with major implications for global food security and economic stability. For example, a study by Zhao et al. (2017) reported that global maize yields decreased by an average of 10% during the mid-twentieth century due to the combined impacts of eCO_2_ and heat stress. Projections for wheat are similarly concerning, with estimates suggesting yield declines of up to 15% by 2050 under scenarios of elevated CO_2_ and increased temperatures (Jayawardena et al. 2020). These findings highlight the urgency of developing robust adaptation strategies to mitigate the adverse effects of climate change on agriculture, particularly in regions already experiencing food insecurity.

Additionally, eCO_2_ may impact on other critical factors, such as crop quality and resistance to pests and diseases. Elevated CO_2_ concentrations have been linked to changes in plant tissue composition, including higher carbon-to-nitrogen ratios, which can reduce the nutritional quality of crops and increase susceptibility to certain pests and pathogens (Abdelhakim et al. 2022). For example, crops grown under eCO_2_ may become more susceptible to pest infestations, such as aphid outbreaks in soybean and cotton, due to changes in plant tissue composition and secondary metabolites (Zavala et al. 2008). Research indicates that higher CO_2_ can decrease the expression of plant defence-related genes, making crops like lima beans (*Phaseolus lunatus*) more vulnerable to aphids (Roden and Ballhorn 2021). Furthermore, a meta-analysis highlighted that eCO_2_ can enhance herbivore performance and pest population growth, leading to increased herbivory pressure (Zhang et al. 2020). These findings raise concerns about the broader implications of eCO_2_ for crop health, food quality, and pest management in future agricultural systems. Despite these insights, there remains a notable gap in research regarding the long-term impacts of eCO_2_ on plant resistance to a wider range of diseases and pathogens, necessitating further exploration in this critical area.

We have indicated that experimental controlled environments typically do not account for the complex interactions of multiple stressors such as highly variable weather patterns —conditions that are common in real-world agricultural systems (Long et al. 2006), and this is the case as well for other stressors like poor soil fertility, pests, and diseases. Thus, projections based solely on findings from controlled environments may overestimate the benefits of eCO_2_ on crop productivity, particularly in regions like sub-Saharan Africa or Southeast Asia, where agricultural infrastructure is limited and the capacity for managing these stressors is minimal. It is, therefore, critical that future agricultural models incorporate these complexities when forecasting crop responses to eCO_2_, especially in resource-constrained settings (Tubiello et al. 2007). Relying on projections from FACE or growth chamber experiments could lead to misinformed policy decisions, particularly in developing countries where such conditions will not be present. These regions are unlikely to benefit from the CO_2_ fertilisation effect at the same scale observed in controlled settings, making their populations more vulnerable to the combined effects of eCO_2_, climate variability and biological stressors (Leakey et al. 2009).

The potential for eCO_2_ to improve agricultural productivity must be evaluated holistically, considering the full spectrum of interacting environmental stressors, as well as the potential trade-offs in crop quality and resistance to pests. Future research and agricultural practices must focus on developing climate-resilient crop varieties and management strategies that can sustainably maximise productivity in the face of multiple, simultaneous stressors. Such strategies will be crucial for safeguarding global food security in a rapidly changing climate.

### Implication on global nutrition

Elevated CO_2_ levels have been consistently linked to reductions in crop nitrogen content, with significant implications for global nutrition. A meta-analysis by Taub and Wang (2008) reported that eCO_2_ concentrations often lead to a decrease in nitrogen concentrations in the leaves, stems, and grains of important crops like wheat, rice, and soybeans. Nitrogen is a critical component of amino acids, the building blocks of proteins. The decline in nitrogen content, therefore, directly reduces the protein content in these crops, which can exacerbate protein malnutrition, particularly in regions where populations rely heavily on these staples for their dietary protein intake.

In addition to reducing protein content, eCO_2_ has been associated with a decline in essential micronutrient content. Research by Myers et al. (2014) demonstrated significant reductions in key minerals such as calcium, zinc, iron, and magnesium in staple crops such as wheat, rice, and barley. A global survey by the World Health Organization (WHO) revealed that nearly 17–30% of the world’s population suffers from iron and zinc deficiency, respectively (WHO 2020). The decrease in these minerals in crop produce could amplify the global burden of hidden hunger—micronutrient deficiencies masked by seemingly adequate energy intake, which lead to severe health consequences, including impaired cognitive development, weakened immune systems, and increased susceptibility to infections. Hidden hunger affects over two billion people globally, particularly in low-income regions (Black et al. 2013), and these numbers are expected to rise in light of deteriorating crop nutritional quality under eCO_2_.

Furthermore, eCO_2_ impacts not only micronutrient concentrations but also carbohydrate metabolism in crops. Increased atmospheric CO_2_ stimulation of photosynthesis leads to an accumulation of carbohydrates, including sugars, in plants (Loladze 2014). While this may initially appear to be a benefit in terms of plant biomass production, it has concerning nutritional ramifications. Research has shown that elevated sugar levels in crops, particularly grains and tubers, this may result in individuals consuming similar amounts of food but with significantly higher sugar content, which can contribute to increasing caloric intakes without a corresponding rise in satiety, thereby leading to rising incidences of obesity and related metabolic disorders, such as diabetes. This can be particularly problematic in developed countries, where diets already feature high sugar and carbohydrate consumption. Over the past few decades, there has been a 30% increase in refined carbohydrate intake, contributing to the surge in obesity and diabetes (Hruby and Hu 2015). In the United States, for instance, over 42% of American adults present with obesity, and diabetes affects over 11% of the population (CDC 2022). The healthcare costs associated with diabetes in the USA exceed $327 billion annually, with the incidence of type 2 diabetes steadily rising (Parker et al. 2024).

In addition to macronutrient changes, eCO_2_ influences the synthesis of vitamins and other bioactive compounds in crops. For example, research by Semba et al. (2022) found that eCO_2_ can reduce concentrations of carotenoids, vitamin C, and vitamin E in crops like rice, soybeans, and leafy vegetables. The decline in these vitamins could have profound implications for populations already experiencing inadequate nutrient intake, as deficiencies in these compounds are associated with weakened immune function, poor eyesight, and increased oxidative stress.

Moreover, the COVID-19 pandemic has further strained global food systems, exacerbating food insecurity and malnutrition in vulnerable populations. Disruptions in food supply chains, coupled with economic downturns, have intensified the risk of hidden hunger, particularly in low- and middle-income countries, where access to nutrient-rich foods is limited (UN OLA 2020; Okolie and Ogundeji 2022). The compounded effects of reduced crop nutritional quality under eCO_2_ and the socioeconomic impacts of global crises present formidable challenges to achieving global nutrition security.

The nutritional degradation of crops under eCO_2_, coupled with the rising prevalence of hidden hunger and metabolic disorders, underscores the critical need for comprehensive strategies to address global malnutrition. From protein and mineral deficiencies in developing countries to rising obesity and diabetes in developed nations, the wide-reaching effects of eCO_2_ on global health necessitate urgent attention and action.

## Critical look into the world’s most grown crop—wheat

**Fig. 4:**
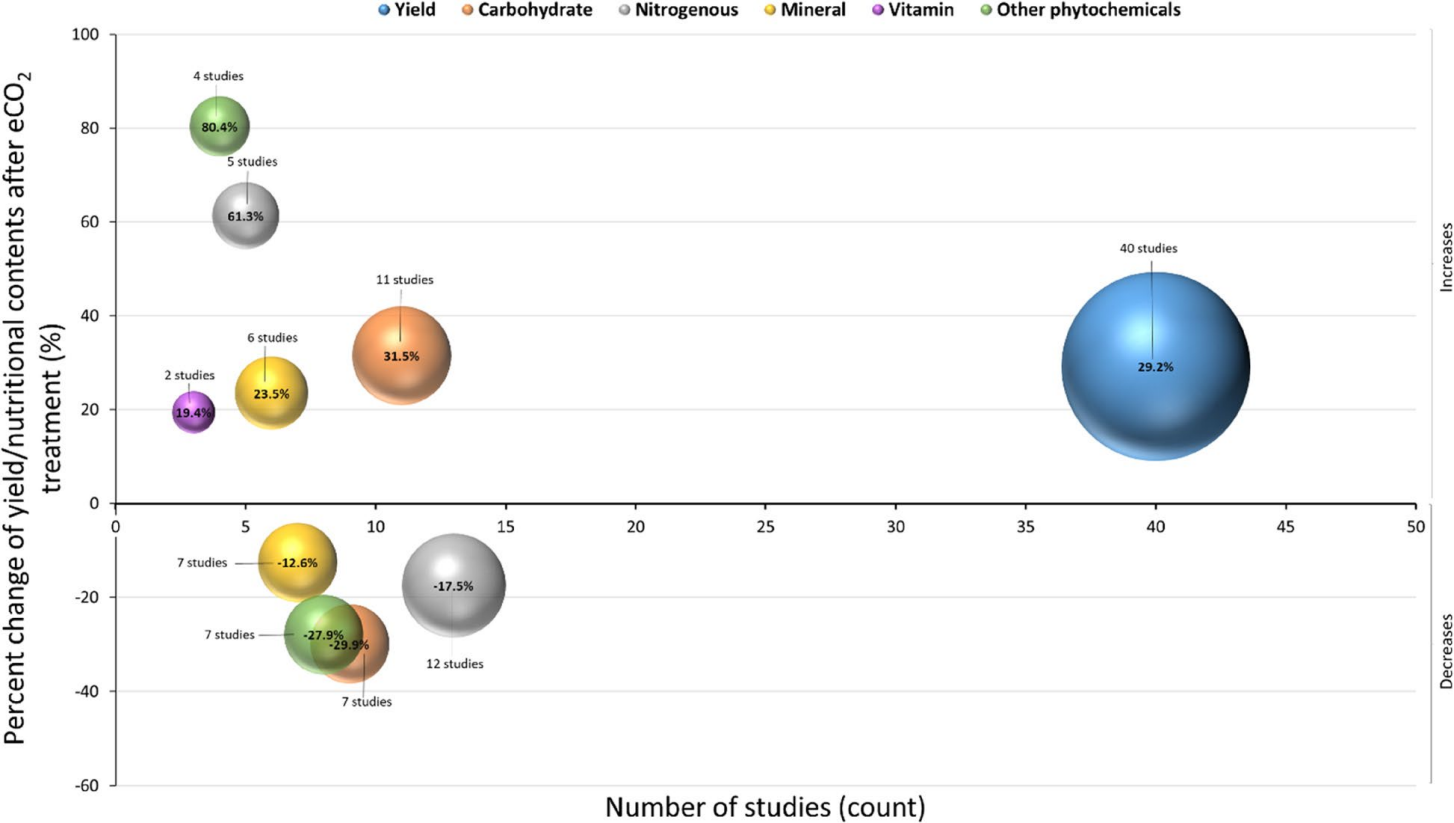
Comparison of the number of experimental studies reporting the quantitative effect of eCO_2_ on yield or nutritional compositions of wheat crop by the change in percentage (%) following a comprehensive literature inquiry conducted on Web of Science, Scopus and PubMed in February 2024. The sizes of the bubbles are equivalent to the number of studies, and the colour depicts the category of plant nutrients

Following an extensive literature search (Web of Science, Scopus and PubMed in February 2024), a minimum of fifty experimental research studies have been found demonstrating the quantitative effects of eCO_2_ levels on wheat yield, biomass, and associated nutritional constituents (see Supplementary material Table 1). It is observed that since approximately 1976, when the investigation into this area began, the central focus had been on yields or grain biomass; however, in the past two decades, there has been a shift to analysing eCO_2_ effects on nutritional composition, such as minerals, vitamins, polyphenols, carbon and nitrogenous constituents (Alsherif and AbdElgawad 2023). Supplementary material Table 1 elaborates on the eCO_2_ effects on the growth and nutritional compositions of wheat grains/crops; all other environmental conditions, such as light intensity, temperature, moisture and soil nutrients, are kept naturally constant for optimum plant survival. Forty studies from the database search have correlated the increase of atmospheric CO_2_ with heightened grain yield, plant dry/fresh biomass, and overall plot yield, demonstrating an average increase of 29.2% (Fig. 4) (Ulfat et al. 2021a; Sayed et al. 2022). Notably, no study within this corpus reported a decrease in yields attributable to eCO_2_, collectively asserting its likelihood to influence yields under controlled conditions and FACE experiments. In particular, yield increases ranged from 5.4% to 78.6% at eCO_2_ levels of 750–1000 ppm compared to control crops at 350–400 ppm (Ulfat et al. 2021b). Similarly, plant biomass/weight increased up to 57.4%. These results indicate that eCO_2_ enhances photosynthesis, leading to greater carbohydrate production and accumulation, ultimately translating into increased biomass and yield (Zhao et al. 2017). The surge in carbon influx also promotes resource allocation to various plant parts, optimising both growth and reproductive output (Zhao et al. 2017).

In addition to yield, eleven studies elaborated on the capacity of eCO_2_ to increase concentrations of various carbohydrate components, including fructose, glucose, maltose, amylose, starch, fructan, and raffinose, with an average rise of 31.5% (Bourgault et al. 2016; Ulfat et al. 2021a). Some studies even recorded increases as high as 199% (Chang et al. 2023). Other carbohydrates, such as amylopectin and starch, showed lower increases, ranging between 1.2% and 20.8% (Wei et al. 2024). These findings support the idea that eCO_2_ stimulates sugar metabolism pathways, impacting carbohydrate allocation and utilisation in plants (Panozzo et al. 2019). However, seven studies reported reductions in sugar content, particularly in fructose, glucose, and sucrose, averaging a 29.9% decrease. These reductions were primarily attributed to varietal disparities in wheat (Chang et al. 2023). Specific cultivars like *Triticum aestivum* L. cv. Shimai 15, cv. Aria, and *Triticum durum* var. Sula exhibited notable reductions in sugar content, particularly fructose, glucose, and fructose (Jauregui et al. 2015). Nitrogenous compounds also showed variable responses to eCO_2_. Five studies indicated an average increase of 61.3% in nitrogenous content, including compounds like glutathione, agmatine, and alanine (Yilmaz et al. 2017). Some studies even reported a doubling or quadrupling of catalase and putrescine levels due to eCO_2_, attributed to differences in wheat cultivars and genetic factors (Abdelhakim et al. 2022; Alsherif and AbdElgawad 2023). However, twelve studies countered this trend, reporting an average reduction of 17.5% in nitrogenous constituents, particularly total protein and nitrogen concentrations (Jayawardena et al. 2020). Several mechanisms explain this decrease, including protein dilution as plants grow larger, the reduced abundance of the key protein RuBisCO, and the inhibition of nitrate conversion into proteins (Fitzgerald et al. 2016). Additionally, increased rhizosphere carbon enrichment under eCO_2_ can impose greater limitations on nitrogen availability to plants (Fitzgerald et al. 2016).

Micronutrient responses of wheat plant to eCO_2_ have also been mixed. Six studies documented an average increase of 23.5% in mineral contents such as sulfur, phosphorus, potassium, and magnesium (Abdelhakim et al. 2022). In contrast, seven studies reported an average decrease of 12.6% in other minerals, particularly in iron, zinc, calcium, and manganese, with reductions reaching up to 66.8% under eCO_2_ concentrations exceeding 700 ppm (Panozzo et al. 2019). These fluctuations in mineral content are influenced by factors such as root length, soil nutrient availability, and the competitive uptake of ions like nitrate and phosphate at root absorption sites (Loladze 2014). Differences in study design may have contributed to these varying findings, including variations in experimental setups, such as growth conditions, the duration of exposure to eCO_2_, and the specific plant species or cultivars studied. Additionally, discrepancies in the nutrient composition of the soils used and the methodologies for measuring nutrient content could further explain the observed differences in micronutrient responses to eCO_2_. Limited research has explored the impact of eCO_2_ on vitamins, but two studies revealed an average increase of 19.4% in α-tocopherols, ascorbate, and other antioxidants (Yilmaz et al. 2017). Increases ranging from 1.8% to 43.3% in vitamin content were attributed to eCO_2_ exposure, suggesting a link between secondary chemical synthesis and eCO_2_-related changes (Alsherif and AbdElgawad 2023). While fewer studies have investigated vitamins, it is anticipated that eCO_2_ could negatively impact certain nutritional elements, particularly nitrogenous compounds (Ziska 2022). Phytochemical responses to eCO_2_ have shown substantial variability. Four studies observed an average increase of 80.4% in phytochemicals such as carotenoids, flavonoids, and polyphenols (Alsherif and AbdElgawad 2023). The concentration of specific compounds like chlorogenic acid and salicylic acid tripled in wheat exposed to eCO_2_, while others, such as malate and oxaloacetate, showed reductions of up to 73% (Jauregui et al. 2015). The synthesis of secondary metabolites like flavonoids may be linked to the surplus carbohydrates produced under eCO_2_, utilising the shikimic acid pathway (Abdelhakim et al. 2022). However, seven studies reported an average decrease of 27.9% in phytochemicals, suggesting that varietal differences could play a significant role in these outcomes (Yilmaz et al. 2017). While eCO_2_ generally promotes wheat yield and biomass, its effects on nutritional composition are mixed. Although eCO_2_ tends to enhance carbohydrate accumulation, it may reduce nitrogenous compounds, minerals, and certain phytochemicals. Understanding these dynamics is crucial as elevated CO_2_ levels continue to alter global agriculture, potentially affecting both crop productivity and nutritional quality.

Wheat demonstrates various morphological changes under eCO_2_ conditions, which influence its growth and productivity. Elevated CO_2_ levels stimulate leaf area expansion, root biomass, and overall tiller production, enhancing the plant’s capacity for photosynthesis and resource acquisition (Abdelhakim et al. 2022). Studies show that eCO_2_ promotes the elongation of wheat stems and increases leaf thickness, primarily due to enhanced cell division and enlargement, which may support greater carbohydrate storage in the shoot tissues (Yilmaz et al. 2017). Additionally, root morphology in wheat adapts to eCO_2_ through increased root length and branching, which improves water and nutrient uptake from the soil, particularly under limited nutrient conditions (Jayawardena et al. 2020). These morphological adjustments contribute to a more extensive root system, which supports the plant’s resilience to intermittent drought stress, often associated with future climate scenarios (Lenka et al. 2021). On a physiological and biochemical level, eCO_2_ affects wheat’s photosynthetic capacity, antioxidant activity, and nutrient composition. Increased CO_2_ availability boosts the efficiency of photosynthesis by reducing photorespiration, particularly in C3 crops like wheat (Prakash et al. 2017). This enhancement results in increased production of sugars and carbohydrates within the plant, which may be stored or utilized for growth, though it often coincides with a decline in protein and micronutrient concentrations in wheat grains (Alsherif and AbdElgawad 2023). Wheat under eCO_2_ also exhibits higher levels of antioxidants such as superoxide dismutase and catalase, which help mitigate oxidative stress induced by environmental factors (Sayed et al. 2022). While this can enhance tolerance to abiotic stressors, the biochemical trade-off includes reduced concentrations of essential minerals such as zinc and iron, posing potential challenges for nutritional quality (Loladze 2014). Collectively, these physiological and biochemical shifts suggest that, although eCO_2_ can improve wheat biomass and stress tolerance, its impact on nutrient density may have adverse implications for food security and health.

## Towards future directions and research needs

Despite some potential benefits of eCO_2_ for crop yields, several challenges and limitations need to be addressed. Crop responses to eCO_2_ vary widely based on species, cultivar, and environmental conditions, making it challenging to generalise findings across different agricultural systems (Abdelhakim et al. 2021). The relationship between eCO_2_, temperature, precipitation, and nutrient availability can modulate crop responses and complicate predictions. Moreover, the long-term impacts of eCO_2_ on soil carbon losses, overall soil health, microbial communities, and other ecosystem functioning remain poorly understood (Chang et al. 2023). Additionally, there are concerns about the potential for eCO_2_ to exacerbate weed and pest pressures, which could offset any yield gains in some cropping systems (Vico and Porporato 2011).

Further research is warranted in several areas to enhance our comprehension of the impacts of eCO_2_ on crop yields and inform adaptation strategies. Firstly, there is a need for holistic and inclusive long-term experiments that encompass diverse cropping systems, climates, and management practices to capture the complex interactions between eCO_2_ and other environmental factors (Jin et al. 2019). Secondly, establishing a cohesive chain of connections between crop breeders, long-term field trials, harvest analysis, product (such as grain) evaluation, and post-harvest or storage nutrient assessment is crucial. Lastly, integrated modelling frameworks that account for biophysical, agronomic, and socio-economic factors are essential for projecting the implications of eCO_2_ on food security, farm productivity, and global food systems (Campbell-Lendrum et al. 2023).

Additionally, studies focusing on the nutritional quality, contents, digestibility and bioavailability, post-harvest traits, and market value of crops exposed to eCO_2_ conditions are necessary to assess the broader implications for health and nutrition, including the impact on animal farming and production for human consumption (Ziska 2022). Finally, efforts to ameliorate the adverse effects of climate change, including cutting back on greenhouse gas emissions and adopting sustainable agricultural practices, are essential for ensuring the resilience and sustainability of agricultural processes in a climatically shifting environment (Shah et al. 2024).

The findings from the combined effect of eCO_2_, heat and drought stressors underscore the pressing need for adaptation mechanisms to mitigate the adverse effects of climate change on agricultural systems, including improved crop varieties, sustainable water management practices, and resilient agricultural policies (Lenka et al. 2021). Addressing the complex interactions among eCO_2_, heat, and drought stress is essential for accurately projecting future crop yields, enhancing global agricultural productivity and maintaining a quality food supply in our changing environment.

## Conclusion

Increased photosynthetic carbon assimilation due to eCO_2_ exposure engenders higher carbohydrate production and accumulation, sometimes translating into greater biomass, yield and carbohydrate production under favourable conditions. These changes stimulate growth and resource allocation to various plant parts, but can also result in a decrease in protein, nitrogen, and certain sugars, as well as reduced nutrient absorption due to impacts on root morphology and soil interactions. While eCO_2_ can improve water use efficiency (WUE) and stimulate photosynthesis in both C3 and C4 plants, these benefits are dependent on environmental factors and plant-specific traits. Despite the potential for higher yields, the overall impact on crop nutritional quality is concerning, as elevated CO2 can reduce concentrations of critical nutrients, exacerbating malnutrition and hidden hunger, particularly in developing countries.

Additionally, increased carbohydrate levels, particularly sugars, pose a health risk in developed countries, contributing to obesity and diabetes. This dual burden of malnutrition—hidden hunger in some regions and overnutrition in others—highlights the complex challenges posed by eCO_2_ on global health. Moreover, the complex interplay between eCO_2_, heat, drought, and other climatic stressors adds formidable challenges in predicting future crop yields and ensuring global food security. Given these realities, addressing the challenges posed by eCO_2_ and its interactions with climatic stressors requires interdisciplinary collaboration to develop resilient agricultural systems that not only ensure adequate yields but also preserve nutritional quality. By combining advances in climate modelling, crop breeding, and sustainable agronomic practices, future food systems can better balance productivity with nutrient-rich outputs, safeguarding global food security and health.

## Supplementary Information

Supplementary Material 1: Table 1. Effect of eCO_2_ on wheat yield and nutritional contents over the last five decades. Only experimental research and experiments that reported data on the impact of eCO_2_ in isolation were included. (Apel 1976; Balouchi et al. 2009; Chaudhuri et al. 1990; Dijkstra et al. 1999; Fangmeier et al. 1996; Gifford 1979; Gifford and Morison 1993; Goudriaan and Ruiter 1983; Grashoff et al. 1995; Högy et al. 2009; Högy et al. 2013; Izaurralde et al. 2003; Kendall et al. 1985; Kimball et al. 2002; Koch 2019; Li et al. 2001; Macabuhay et al. 2018; Manoj-Kumar et al. 2012; McKee et al. 1994; McKee et al. 1997; Mitchell et al. 1996; Monje and Bugbee 1998; UN OLA 2010; Pinter et al. 2000; Pleijel et al. 2000; Reuveni and Bugbee 1997; Rogers et al. 1998; Singh et al. 2013; Sionit et al. 1981; Veisz et al. 1996; Weigel et al. 1994; Wolf 1996).

## Data Availability

All data generated or analysed during this study are included in this published article and its supplementary information file.

## Abbreviations

CO_2_: Atmospheric carbon dioxide
eCO_2_: Elevated carbon dioxide
C3: Plants that produce a three-carbon compound (3-phosphoglyceric acid) through the Calvin-Benson cycle
C4: Plants whose first product of carbon fixation is a 4-carbon compound
SDG2: Sustainable Development Goal 2
COVID-19: Coronavirus disease of 2019
RuBisCO: The enzyme ribulose-1,5-bisphosphate carboxylase/oxygenase
CBB: Calvin–Benson–Bassham cycle
RuBP: Ribulose-1,5-bisphosphate
ATP: Adenosine triphosphate
NADPH: Nicotinamide adenine dinucleotide phosphate
PEPC: Phosphoenolpyruvate carboxylase
FACE: Free-air CO_2_ enrichment
ppm: Parts per million
WUE: Water Use Efficiency
