# Supplementary material for: Current impacts of elevated CO2 on crop nutritional quality: a review using wheat as a case study
Source: Stress Biol. 2025 May 8;5(1):34. doi: 10.1007/s44154-025-00217-w (PMC12061828; doi:10.1007/s44154-025-00217-w)
Supplement: Supplementary file 1 — Supplementary Material 1: Table 1. Effect of eCO2 on wheat yield and nutritional contents over the last five decades. Only experimental research and experiments that reported data on the impact of eCO2 in isolation were included. (Apel 1976; Balouchi et al. 2009; Chaudhuri et al. 1990; Dijkstra et al. 1999; Fangmeier et al. 1996; Gifford 1979; Gifford and Morison 1993; Goudriaan and Ruiter 1983; Grashoff et al. 1995; Högy et al. 2009; Högy et al. 2013; Izaurralde et al. 2003; Kendall et al. 1985; Kimball et al. 2002; Koch 2019; Li et al. 2001; Macabuhay et al. 2018; Manoj-Kumar et al. 2012; McKee et al. 1994; McKee et al. 1997; Mitchell et al. 1996; Monje and Bugbee 1998; UN OLA 2010; Pinter et al. 2000; Pleijel et al. 2000; Reuveni and Bugbee 1997; Rogers et al. 1998; Singh et al. 2013; Sionit et al. 1981; Veisz et al. 1996; Weigel et al. 1994; Wolf 1996). [file 44154_2025_217_MOESM1_ESM.pdf]

Supplementary Data

Table 1: Effect of eCO_2 on wheat yield and nutritional contents over the last five decades. Only experimental research and experiments that reported data on the impact of eCO_2 in isolation were included.

Category	Quality parameter	eCO_2 effect	Percentage change from ambient CO_2 (and citation)
Yield	Grain yield/weight (g) (g.plant^{-1}) (g.m^{-2})DW (kg.ha^{-1}) or (kg.m^{-2})	Increase	5.4-13.3% (McKee & Woodward 1994), 6.3% (Cai *et al*. 2016), 9.3-78.6% (Ulfat *et al*. 2021[a]), 10.4-10.8% (Högy *et al*. 2013), 12.7% (Mitchell *et al*. 1996), 13.3% (Monje & Bugbee 1998), 13.7% (Veisz *et al*. 1996), 14.2-16.7% (Bourgault *et al*. 2016), 14.7% (Pinter *et al*. 2000), 15% (Singh *et al*. 2013), 15% (Lenka *et al*. 2021), 16.5% (Kendall *et al*. 1985), 17-23% (Wei *et al*. 2024), 18.1% (Pleijel *et al*. 2000), 18.7-24% (Sayed *et al*. 2022), 20.1% (Balouchi *et al*. 2009), 23.5% (Grashoff *et al*. 1995), 24.8% (Li *et al*. 2001), 25% (Reuveni & Bugbee 1997), 25.5% (Gifford 1979), 28.3% (McKee *et al*. 1997), 30.1% (Chaudhuri *et al*. 1990), 30.5% (Dijkstra *et al*. 1999), 30.6% (Chavan *et al*. 2019), 32.4% (Manoj-Kumar *et al*. 2012), 36.4-49.7% (Abdelhakim *et al*. 2022), 45.3- 51.6% (Prakash *et al*. 2017), 34.1% (Grashoff *et al*. 1995), 36.2% (Gifford & Morison 1993), 36.5% (Apel 1976), 37% (Fitzgerald *et al*. 2016), 39% (Macabuhay *et al*. 2018), 41.4% (Reuveni & Bugbee 1997), 49.6% (Gifford 1979), 50.4% (Ulfat *et al*. 2021a), 56% (Fitzgerald *et al*. 2016), 59.6- 62.1% (Sionit *et al*. 1981)
	Plant biomass (g) or weight (g plant^{-1})		47.5% (Jayawardena *et al*. 2020), 6.9- 17.9% (Abdelhakim *et al*. 2022), 19.3- 57.4% (Sayed *et al*. 2022)
	Plot yield or weight (g.plot^{-1}) (g.pot^{-1}) or (kg.m^{-3})		17.9- 26.9% (Weigel *et al*. 1994), 27% (Fangmeier *et al*. 1996), 33% (Izaurralde *et al*. 2003), 2.5- 27.3% (Rogers *et al*. 1998), 59.4% (Goudriaan & de Ruiter 1983), 74% (Wolf *et al*. 1996), 1% (Kimball *et al*. 2002)
Carbohydrate contents	Fructose (mg.g^{-1}) or (nmol mg^{-1}) DW/FW	Increase	14.3-116.7% (Abdelhakim *et al*. 2021), 15.1 (Högy *et al*. 2009), 18.5 (Högy *et al*. 2013), 29.3- 58% (Ulfat *et al*. 2021[b])
	Glucose (mg.g^{-1}) DW/FW		10-12.6% (Högy *et al*. 2009), 11-161.5% (Ulfat *et al*. 2021[a]), 15-106.7% (Abdelhakim *et al*. 2021), 32.1% (Chang *et al*. 2023), 199% (Abdelhakim *et al*. 2022)
	Maltose (g.m^{-2}) DW		1.6% (Högy *et al*. 2013), 15.7% (Högy *et al*. 2009)
	Sucrose (mg g^{-1}) DW/FW		5.2-105.1% (Ulfat *et al*. 2021[a]), 11.6% (Högy *et al*. 2009), 13% (Högy *et al*. 2013), 32% (Abdelhakim *et al*. 2021)
	Amylopectin (mg.g^{-1})		1.2- 4.2% (Wei *et al*. 2024)
	Amylose (mg.g^{-1})		6.5-11% (Wei *et al*. 2024)
	Starch (mg.g^{-1}) or (g.m^{-2}) DW		5.2% (Högy *et al*. 2013), 36.1% (Chang *et al*. 2023), 63.9% (Jauregui *et al*. 2015)
	Fuctan (g.m^{-2})DW		7.6% (Högy *et al*. 2009), 33.2% (Högy *et al*. 2013)
	Raffinose (g.m^{-2})DW		14.8% (Högy *et al*. 2009), 20.8% (Högy *et al*. 2013)
	Total non-structural carbohydrates (g.m^{-2}) or (mg.g^{-1})DW		3.2% (Chang *et al*. 2023), 5.9% (Högy *et al*. 2013), 14% (Högy *et al*. 2009)
	Total starch content (mg.g^{-1})		3.3-4.7% (Wei *et al*. 2024)
	Water soluble carbohydrate (mg.g^{-1})		3.5- 3.9% (Panozzo *et al*. 2019), 61.2% (Bourgault *et al*. 2016)
	Starch (mg.g^{-1}) or (g.m^{-2}) DW	Decrease	-2.5% (Högy *et al*. 2009), -19.3 -46% (Abdelhakim *et al*. 2021)
	Fructose (mg.g^{-1}) or (nmol mg^{-1}) DW/FW		-6.4% (Jauregui *et al*. 2015), -20.5-58.2% (Abdelhakim *et al*. 2021), -39% (Ulfat *et al*. 2021[a]), -62.4% (Chang *et al*. 2023)
	Glucose (mg.g^{-1}) DW/FW		-17.4% (Abdelhakim *et al*. 2021), -43.6% (Jauregui *et al*. 2015), -74.1% (Abdelhakim *et al*. 2022)
	Sucrose (mg g^{-1}) DW/FW		-1.5-54.5% (Abdelhakim *et al*. 2021), -17.4% (Chang *et al*. 2023), -20.2% (Jauregui *et al*. 2015)

	Soluble sugar content (mg.g^{-1}) DW		-29.2% (Chang *et al*. 2023), -35.7% (Balouchi *et al*. 2009)
Nitrogenous contents	catalase (mg.g^{-1}) FW	Increase	78% (Alsherif & AbdElgawad 2023)
	Dehydroascorbate reductase (mg.g-1) FW		176% (Alsherif & AbdElgawad 2023)
	Glutathione peroxidase (mg.g-1) FW		81.3% (Alsherif & AbdElgawad 2023)
	Glutathione reductase (mg.g-1) FW or (µ.mg^{-1})		13.9% (Yilmaz *et al*. 2017), 49.1% (Alsherif & AbdElgawad 2023)
	Peroxidase (mg.g-1) FW		27% (Alsherif & AbdElgawad 2023)
	Spermidine (pmol.mg-1) DW		17.3- 64.6% (Abdelhakim *et al*. 2022)
	Norspermidine (pmol.mg^{-1}) DW		112.1% (Abdelhakim *et al*. 2022)
	1,3-diaminopropane (pmol.mg^{-1}) DW		12.2% (Abdelhakim *et al*. 2022)
	2-aminoapidic acid (pmol.mg^{-1}) DW		25% (Abdelhakim *et al*. 2022)
	Putrescine (pmol.mg^{-1}) DW		246% (Abdelhakim *et al*. 2022)
	Agmantine (pmol.mg^{-1}) DW		18.6% (Abdelhakim *et al*. 2022)
	Alanine (pmol.mg-1) DW		11- 19% (Abdelhakim *et al*. 2022)
	Arginine (pmol.mg-1) DW		20% (Abdelhakim *et al*. 2022)
	Asparagine (pmol.mg-1) DW		49.5- 106.5% (Abdelhakim *et al*. 2022)
	Aspartic acid (pmol.mg-1) DW		28.4% (Abdelhakim *et al*. 2022)
	Cysteine (pmol.mg-1) DW		23.9% (Abdelhakim *et al*. 2022)
	Glutamate (mg.g^{-1})		5.2% (Jauregui *et al*. 2015)
	Histidine (pmol.mg-1) DW		48.9% (Abdelhakim *et al*. 2022)
	Lysine (pmol.mg-1) DW		45.7- 97.8% (Abdelhakim *et al*. 2022)
	Methionine (pmol.mg-1) DW		15.2% (Abdelhakim *et al*. 2022)
	N-acetyl glutamate (pmol.mg-1) DW		0.6- 9% (Abdelhakim *et al*. 2022)
	Tryptophan (pmol.mg-1) DW		402% (Abdelhakim *et al*. 2022)
	Tyrosine (pmol.mg-1) DW		64.4% (Abdelhakim *et al*. 2022)
	β -alanine (pmol.mg-1) DW		33% (Abdelhakim *et al*. 2022)
	β -aminobutyric acid (pmol.mg-1) DW		40- 54% (Abdelhakim *et al*. 2022)
	γ -aminobutyric acid (pmol.mg-1) DW		4.7% (Abdelhakim *et al*. 2022)
	Histamine (pmol.mg-1) DW		34- 85.2% (Abdelhakim *et al*. 2022)
	Citrulline (pmol.mg-1) DW		85.1% (Abdelhakim *et al*. 2022)
	N-acetylornithine (pmol.mg-1) DW		100.2% (Abdelhakim *et al*. 2022)
	Ornithine (pmol.mg-1) DW		5% (Abdelhakim *et al*. 2022)
	Soluble protein (mg.g-1) DW		19% (Chang *et al*. 2023)
	Spermidine (pmol.mg-1) DW	Decrease	-40.3- 64.6% (Abdelhakim *et al*. 2022)
	Superoxide dismutase (mg.g^{-1}) FW		-1.3% (Alsherif & AbdElgawad 2023)
	1,3-diaminopropane (pmol.mg^{-1}) DW		-52.8% (Abdelhakim *et al*. 2022)
	Putrescine (pmol.mg^{-1}) DW		-6.3% (Abdelhakim *et al*. 2022)
	Norspermidine (pmol.mg^{-1}) DW		-41.7% (Abdelhakim *et al*. 2022)
	2-aminoapidic acid (pmol.mg^{-1}) DW		-45.5% (Abdelhakim *et al*. 2022)
	Agmantine (pmol.mg^{-1}) DW		-22.3% (Abdelhakim *et al*. 2022)

	Alanine (g.m^{-2}) or (mg.g^{-1}) DW		-5.3% (Hogy *et al*. 2009), -7% (Hogy *et al*. 2013), -18.8% (Jauregui *et al*. 2015)
	Dehydroascorbate reductase (µ.mg^{-1})		-4.5% (Yilmaz *et al*. 2017)
	Ascorbate peroxidase (µ.mg^{-1})		-0.8% (Yilmaz *et al*. 2017)
	Gliadin (g.m^{-2}) or (mg.g^{-1}) DW		-8.1% (Högy *et al*. 2009), -13.3% (Högy *et al*. 2013),
	Glutathione-S-transferase (nmol/min.mg)		-31.6% (Alsherif & AbdElgawad 2023)
	Gluten (g.m^{-2}) or (mg.g^{-1}) DW		-7.2% (Högy *et al*. 2009), -11.3% (Högy *et al*. 2013),
	Glutenin (g.m^{-2}) or (mg.g^{-1}) DW		-5.5% (Högy *et al*. 2009), -5.7% (Högy *et al*. 2013),
	Metallothioneins (nmol.g^{-1}) FW		-17.5% (Alsherif & AbdElgawad 2023)
	Monodehydroascorbate reductase (µ.mg^{-1})		-50.1% (Yilmaz *et al*. 2017)
	Cadaverine (pmol.mg^{-1}) DW		-25-45.45% (Abdelhakim *et al*. 2022)
	Arginine (g.m^{-2}) or (pmol.mg^{-1}) DW		-5.5% (Högy *et al*. 2009), -5.6% (Högy *et al*. 2013), -33.3% (Abdelhakim *et al*. 2022)
	Asparagine (g.m^{-2}) or (pmol.mg^{-1}) DW		-5.2% (Högy *et al*. 2009), -5.5% (Jauregui *et al*. 2015), -8.2% (Högy *et al*. 2013), -41.4% (Abdelhakim *et al*. 2022)
	Cysteine (g.m^{-2}) or (pmol.mg^{-1}) DW		-4.2% (Högy *et al*. 2013), -7.4% (Högy *et al*. 2009), -42.5% (Abdelhakim *et al*. 2022)
	Glutamic acid (g.m^{-2}) or (pmol.mg^{-1}) DW		-6.2% (Högy *et al*. 2013), -10.7% (Högy *et al*. 2009), -16.4-31.4% (Abdelhakim *et al*. 2022)
	Gamma-aminobutyric acid (mg.g^{-1})		-28.1% (Jauregui *et al*. 2015)
	Glutamine (mg.g^{-1}) or (pmol.mg^{-1}) DW		-8.6- 20% (Abdelhakim *et al*. 2022), -64.8% (Jauregui *et al*. 2015)
	Glycine (g.m^{-2})		-5.7% (Högy *et al*. 2013), -8.6% (Högy *et al*. 2009)
	Histidine (g.m^{-2})		-5.1% (Högy *et al*. 2009), -5.5% (Högy *et al*. 2013), -60.8% (Abdelhakim *et al*. 2022)
	Homoarginine (pmol.mg-1) DW		-7—28.9% (Abdelhakim *et al*. 2022)
	Isoleucine (g.m^{-2})		-7.4% (Högy *et al*. 2013), -8.7% (Högy *et al*. 2009)
	Leucine (g.m^{-2})		-6.1% (Högy *et al*. 2013), -8.6% (Högy *et al*. 2009)
	Lysine (g.m^{-2})		-5.4% (Högy *et al*. 2013), -7.1% (Högy *et al*. 2009)
	Methionine (g.m^{-2}) or pmol.mg^{-1}) DW		-6.1% (Högy *et al*. 2009), -8.3% (Högy *et al*. 2013), -32% (Abdelhakim *et al*. 2022)
	Phenylalanine (g.m^{-2}) or pmol.mg^{-1}) DW		-5-22.7% (Abdelhakim *et al*. 2022), -7.6% (Högy *et al*. 2009), -8.4% (Högy *et al*. 2013)
	Proline (µg.g^{-1}) (g.m^{-2}) (mg.g^{-1})or pmol.mg^{-1}) DW		-4-65.2% (Abdelhakim *et al*. 2022), -7.4% (Högy *et al*. 2013), -9.8% (Högy *et al*. 2009), -6.8% (Balouchi *et al*. 2009), -13.8% (Jauregui *et al*. 2015)
	Serine (g.m^{-2}) or pmol.mg^{-1}) DW		-7.1% (Högy *et al*. 2009), -7.3% (Högy *et al*. 2013), -20.1-25.1% (Abdelhakim *et al*. 2022)
	Threonine (g.m^{-2}) or pmol.mg^{-1}) DW		-5.3% (Högy *et al*. 2009), -6.8% (Högy *et al*. 2013), -26.9-34.8% (Abdelhakim *et al*. 2022)
	Tryptophan (g.m^{-2}) or pmol.mg^{-1}) DW		-5.3% (Högy *et al*. 2009), -6% (Högy *et al*. 2013), -6.7% (Abdelhakim *et al*. 2022)
	Tyrosine (g.m^{-2}) or pmol.mg^{-1}) DW		-7.9% (Högy *et al*. 2013), -8.9% (Högy *et al*. 2009), -10.1% (Abdelhakim *et al*. 2022)
	Valine (g.m^{-2}) or pmol.mg^{-1}) DW		-6.6% (Högy *et al*. 2013), -8.1% (Högy *et al*. 2009), -15 -27% (Abdelhakim *et al*. 2022)
	β -alanine (pmol.mg^{-1}) DW		-26.4% (Abdelhakim *et al*. 2022)
	γ -aminobutyric acid (pmol.mg^{-1}) DW		-40.3% (Abdelhakim *et al*. 2022)
	Citrulline (pmol.mg^{-1}) DW		-47% (Abdelhakim *et al*. 2022)
	N-acetylornithine (pmol.mg^{-1}) DW		-30.2% (Abdelhakim *et al*. 2022)
	Ornithine (pmol.mg^{-1}) DW		-36.5% (Abdelhakim *et al*. 2022)
	Protein content (µg.g^{-1}) or (mg.g^{-1}) FW		-7.4-8.5% (Wei *et al*. 2024), -8.9-17.1% (Myers *et al*. 2014), -18% (Balouchi *et al*. 2009), -19.1% (Jayawardena *et al*. 2020), -19.5% (Jauregui *et al*. 2015)
	Grain & flour protein (kg.N)		-5% (Kimball *et al*., 2002)

Vitamin contents	Alpha tocopherols (mg.g^{-1}) FW	Increase	43.3% (Alsherif & AbdElgawad 2023)
	Ascorbate (mg.g^{-1}) FW		18% (Alsherif & AbdElgawad 2023)
	Ascorbate peroxidase (mg.g^{-1}) FW		14.3% (Alsherif & AbdElgawad 2023)
	Ascorbic acid (mg.g^{-1}) FW		1.9% (Yilmaz *et al*. 2017)
Mineral contents	Phosphorous concentration in grain and leaf (mg.g^{-1})DW	Increase	8.7- 23.3% (Sayed *et al*. 2022)
	Aluminium (g.m^{-2}) DW		8.8% (Högy *et al*. 2013)
	Boron (g.100g^{-1}) or (g.m^{-2}) DW		27.2% Högy *et al*. (2013), 80.9% (Jauregui *et al*. 2015)
	Chloride (pmol mg^{-1}) DW		1.3- 25.7% (Abdelhakim *et al*. 2022)
	Chromium (g.m^{-2}) DW		4.6% (Högy *et al*. 2009)
	Copper (mg.kg^{-1})		14.9% (Yilmaz *et al*. 2017)
	Lead (g.m^{-2}) DW		15% (Högy *et al*. 2009)
	Magnesium (g.100g^{-1})		13.7% (Jauregui *et al*. 2015)
	Molybdenum (g.100g^{-1}) or (g.m^{-2}) DW		35.8% (Jauregui *et al*. 2015), 7.7% (Högy *et al*. 2013), 8.4% (Högy *et al*. 2009)
	Nitrate (pmol.mg^{-1}) DW		127.1% (Abdelhakim *et al*. 2022)
	Phosphate (pmol mg^{-1}) DW		26.6% (Abdelhakim *et al*. 2022)
	Potassium (g.m^{-2}) DW		4.5% (Högy *et al*. 2009)
	Selenium		18.2% (Högy *et al*. 2013)
	Sodium (g.m^{-2}) DW		10.4% (Högy *et al*. 2013), 17% (Högy *et al*. 2009)
	Sulfate (pmol.mg^{-1}) DW		37.3% (Abdelhakim *et al*. 2022)
	Sulfur (g.m^{-2}) DW		2.4% (Högy *et al*. 2013)
	Zinc (mg.kg^{-1}) or (g.m^{-2}) DW		0.7% (Högy *et al*. 2013), 27.4% (Yilmaz *et al*. 2017)
	Cadmium (g.m^{-2}) DW	Decrease	-8.1% (Högy *et al*. 2013), -14.1% (Högy *et al*. 2009)
	Calcium (g.100g^{-1})		-3% (Högy *et al*. 2009), -6.6% (Högy *et al*. 2013), -17.1% (Jauregui *et al*. 2015)
	Chromium (g.m^{-2}) DW		-4.1% (Högy *et al*. 2013)
	Copper (g.100g^{-1}) or (g.m^{-2}) DW		-1.5% (Högy *et al*. 2013), -2.6% (Högy *et al*. 2009), -28.5% (Jauregui *et al*. 2015)
	Iron (g.100g^{-1}) (mg.kg^{-1}) or (g.m^{-2}) DW		-31.9% (Jauregui *et al*. 2015), 9.5% (Högy *et al*. 2009), -5.9% (Högy *et al*. 2013), -1.8% (Yimaz *et al*. 2017), -2.3-8.7% (Myers *et al*. 2014)
	Lead (g.m^{-2}) DW		-9.5% (Högy *et al*. 2013)
	Leaf Nitrogen (g.100g^{-1})		-11.2% (Jauregui *et al*. 2015)
	Magnesium (g.m^{-2}) DW		-1.6% (Högy *et al*. 2009), -2.7% (Högy *et al*. 2013)
	Manganese (g.100g^{-1}) (mg.kg^{-1}) or (g.m^{-2}) DW		-3.9% (Högy *et al*. 2009), -1.5% (Högy *et al*. 2013), -7.2% (Yilmaz *et al*. 2017), -66.8% (Jauregui *et al*. 2015)
	Nickel (g.m^{-2}) DW		-2% (Högy *et al*. 2009), -2.3% (Högy *et al*. 2013)
	Nitrate (pmol mg^{-1}) DW		-8% (Abdelhakim *et al*. 2022), -3.7% (Panozzo *et al*. 2019)
	Phosphate (pmol mg^{-1}) DW		-29.6% (Abdelhakim *et al*. 2022)
	Phosphorous (g.100g^{-1}) or (g.m^{-2}) DW		-0.9% (Högy *et al*. 2009), -1.4 (Högy *et al*. 2013), -16.7% (Jauregui *et al*. 2015)
	Potassium (g.100g^{-1}) or (g.m^{-2}) DW		-1.9% Högy *et al*. (2013), -36.8% Jauregui *et al*. (2015)
	Selenium (g.m^{-2}) DW		-11% (Högy *et al*. 2009)
	Silicon (g.m^{-2}) DW		-6.9% (Högy *et al*. 2009), -10.2% (Högy *et al*. 2013)

	Sulfate (pmol.mg^{-1}) DW		-64.7% (Abdelhakim *et al*. 2022)
	Sulfur (g.100g^{-1}) or (g.m^{-2}) DW		-6.5% (Jauregui *et al*. 2015), -7% (Högy *et al*. 2009)
	Zinc (g.100g^{-1}) or (g.m^{-2}) DW		-49% (Jauregui *et al*. 2015), 2% Högy *et al*. (2009), -9.3-12.3% (Myers *et al*. 2014)
Other phytochemicals	Carotenoid (mg.g^{-1}) (µmol.g^{-1}) or (mg.g^{-1}) FW/DW	Increase	0.1% (Balouchi *et al*. 2009), 4.7% (Alsherif & AbdElgawad 2023), 10% (Abdelhakim *et al*. 2021)
	Ferulic acid (pmol.mg^{-1}) DW		40.7- 55.8% (Abdelhakim *et al*. 2022)
	Chlorogenic acid (pmol.mg^{-1}) DW		480.8% (Abdelhakim *et al*. 2022)
	Flavonoids (mg.g^{-1}) FW		67.9% (Alsherif & AbdElgawad 2023)
	Polyphenol (mg.g^{-1}) FW		9.8% (Alsherif & AbdElgawad 2023)
	Salicylic acid (pmol.mg^{-1}) DW		270% (Abdelhakim *et al*. 2022)
	Chlorophyll b (mg.g^{-1}) FW		50% (Alsherif & AbdElgawad 2023)
	Total chlorophyll (mg.g^{-1}) DW		13% (Abdelhakim *et al*. 2021)
	4-hydroxybenzoic acid (pmol.mg^{-1}) DW		39.2- 68% (Abdelhakim *et al*. 2022)
	Citrate (pmol.mg^{-1}) DW		3.2% (Abdelhakim *et al*. 2022)
	Total glutathione (nmol.g^{-1}) FW		33.3% (Alsherif & AbdElgawad 2023)
	Anthocyanin (µmol.g^{-1}) FW	Decrease	-25.5% (Balouchi *et al*. 2009)
	Chlorogenic acid (pmol.mg^{-1}) DW		-86.9% (Abdelhakim *et al*. 2022)
	Flavonoid 270 (µmol.g^{-1}) FW		-28.7-68.8% (Balouchi *et al*. 2009)
	Phytate		-4.1% (Myers *et al*., 2014)
	phytochelatins (nmol.g^{-1}) FW		-12% (Alsherif & AbdElgawad 2023)
	Salicylic acid (pmol.mg^{-1}) DW		-13-80% (Abdelhakim *et al*. 2022)
	Carotenoid (mg.g^{-1}) DW		-6.9 -9% (Abdelhakim *et al*. 2021)
	Chlorophyll a (mg.g^{-1}) FW		-10.7% (Alsherif & AbdElgawad 2023)
	Chlorophyll a:b (mg.g^{-1}) DW		-1-6.5% (Abdelhakim *et al*. 2021)
	Total chlorophyll (mg.g^{-1}) DW		-1-5.9% (Abdelhakim *et al*. 2021),
	Oxaloacetate (mg.g^{-1})		-39.5% (Jauregui *et al*. 2015)
	p-coumaric acid (pmol.mg^{-1}) DW		-20.7-27% (Abdelhakim *et al*. 2022)
	Malate (mg.g^{-1}) or (pmol.mg^{-1}) DW		-6.3-48% (Abdelhakim *et al*. (2022), -73% (Jauregui *et al*. 2015)
	Citrate (mg.g^{-1}) or (pmol.mg^{-1}) DW		-8.9% (Abdelhakim *et al*. 2022), -52.9% (Jauregui *et al*. 2015)
	Thiobarbituric Acid (nmol.g^{-1}) FW		-15.2% (Yilmaz *et al*. 2017)
	Reduced glutathione (mg.g-1) FW		-12.9% (Alsherif & AbdElgawad 2023)
